# Dosages of swallowing exercises in stroke rehabilitation: a systematic review

**DOI:** 10.1007/s00405-022-07735-7

**Published:** 2022-12-06

**Authors:** Jacinda Choy, Fereshteh Pourkazemi, Caitlin Anderson, Hans Bogaardt

**Affiliations:** 1 Faculty of Medicine and Health, The University of Sydney, Sydney, NSW Australia; 2 HammondCare Braeside Hospital, Sydney, NSW Australia; 3 School of Allied Health Science and Practice, The University of Adelaide, Adelaide, SA Australia

**Keywords:** Deglutition disorders, Stroke, Exercise therapy, Rehabilitation, Speech-language pathology, Systematic review

## Abstract

**Purpose:**

To investigate the dosages of swallowing exercises reported in intervention studies on post-stroke dysphagia through systematic review.

**Methods:**

Five electronic databases were searched from inception until February 2022 with reference tracing of included studies. Studies were included, where adults with post-stroke dysphagia received rehabilitative, behavioural swallowing exercises, pre/post outcomes were reported, and intervention dosage was described in detail, including frequency, intensity, time, and type of exercise. Two reviewers independently screened studies and rated quality using ASHA Levels of Evidence tool. Data was tabulated and narratively described.

**Results:**

54 studies were included with a total 1501 participants. Studies included 28 randomised controlled trials, 8 non-randomised controlled trials, 12 pre/post studies, 3 retrospective case controls and 3 case studies. Results showed inconsistent reporting of intervention dosage, with intensity the least consistently reported dosage component. While swallowing intervention was most commonly provided five times per week for four weeks, there was a wide breadth of type, frequency, intensity and duration of swallowing exercises reported. Dosage under-reporting and variation was particularly observed in “standard care” co-interventions or control groups. Study strengths included following PRISMA guidelines, providing a comprehensive review of swallowing exercise methodology and dosages, and including non-English studies. The limitation was lack of meta-analysis due to the heterogeneity of included studies.

**Conclusions:**

Dosages of swallowing exercises are inconsistently reported and vary significantly in post-stroke dysphagia studies. Results indicate the need for consistent and comprehensive dosage reporting in dysphagia studies, and for further research into evidence-based principles to optimise swallowing exercise dosages.

**Systematic review registration number:**

131294

**Supplementary Information:**

The online version contains supplementary material available at 10.1007/s00405-022-07735-7.

## Introduction

Dysphagia is a common and significant symptom following stroke. Dysphagia, or swallowing difficulties, affect a third to over two thirds of patients after stroke [1, 2]. Dysphagia causes medical complications, including increased hospitalisation, morbidity, and risk of aspiration pneumonia [3]. It is associated with poor psychosocial health outcomes, such as reduced nutrition, hydration and quality of life [4]. Patients with dysphagia have longer lengths of hospital stay and higher healthcare costs [5, 6].

Current management of dysphagia involves compensation and rehabilitation. Compensatory techniques—such as chin tuck or modifying diet and fluid consistencies—enable safe swallowing but do not alter long-term function [7]. Rehabilitative exercises, however, can improve swallowing function and resumption of oral intake or normal food and drink [7]. Rehabilitative exercises can be indirect (motor without swallow) or direct (motor with swallow) [8]. Indirect exercises aim to strengthen muscles involved in swallowing, and include the Shaker head-lift and tongue strengthening exercises [9]. Direct exercises involve the action of swallowing and include the Mendelsohn manoeuvre and effortful swallow [9]. Studies have shown the positive effects of rehabilitative exercises on reducing the severity and symptoms of post-stroke dysphagia [10].

However, there is limited understanding of the optimal way to conduct swallowing exercises, particularly, the optimal dosages of swallowing exercises. Dosage is an important factor which can impact on intervention efficacy and efficiency [11]. According to the American College of Sports Medicine’s (ACSM) FITT framework, dosage consists of Frequency (how often), Intensity (how hard), Time (how long) and Type (what kind) of exercise [12]. Altering or increasing these components of dosage can optimise exercise or intervention outcomes, as seen in sports medicine and stroke rehabilitation [13, 14]. However, there is limited knowledge on what dosages to use for swallowing exercises. Previous scoping and literature reviews have highlighted the paucity of data regarding dosage recommendations in dysphagia intervention [9, 15]. This is reflected in surveys of speech pathologists which indicate variability in the exercises and dosages used to treat dysphagia [16].

The aim of this systematic review was to investigate what dosages of swallowing exercises are reported in studies in post-stroke dysphagia. To our knowledge, only one scoping review has specifically examined the dosages of swallowing exercises [15]. Our review was conducted to systematically update the search with new studies. Given that intervention dosage may vary depending on diagnosis, this review focused on one of the most common causes of acquired dysphagia: stroke [17]. This systematic review aimed to investigate dosage reporting in research and describe current swallowing exercise dosages in intervention studies to guide clinicians when considering dosage prescription. The findings can be used to identify areas for future research in optimising dosage of swallowing exercises to facilitate more cost-effective intervention, increased patient engagement and improved outcomes.

## Methods

This systematic review was conducted according to PRISMA guidelines [18]. Prior to conducting the study, a protocol was registered on PROSPERO (https://www.crd.york.ac.uk/PROSPERO/, registration number: 131294).

### Eligibility criteria

Studies were included if: (i) they included adult participant/s with dysphagia due to stroke, (ii) they examined rehabilitative, behavioural swallowing exercises, (iii) they were a published intervention study, where pre/post outcomes were reported, and (iv) they provided a detailed description of the dosage of the rehabilitative exercise. Only full-text published studies accessible through online databases were included in this review. For the purpose of this review, the ACSM FITT framework was used to specify the minimal elements required in a detailed description of dosage [12]. Dosage description needed to include the frequency (or number) of sessions, intensity or dose (at a minimum, the number of repetitions of each exercise), intervention duration and type of exercise. Stating the type of exercise required the name and reference of a well-known exercise, or a detailed description of the materials, procedures, activities and/or processes involved in the exercise (as per points 1–4 of the Template for Intervention Description and Replication checklist) [19]. Studies were included even if only a subset of participants matched inclusion criteria (e.g., participants with dysphagia due to stroke and other conditions). This review focused on methodology reporting, not intervention effect, so type of outcome measure was not an inclusion criterion. Outcome measures were collected as reported, without limit. Studies in all languages were included. Studies were excluded if they *only* applied passive interventions, such as acupuncture, thermal–tactile stimulation, compensatory strategies, or electrical stimulation (i.e., when not combined with active exercise), as these require different dosages to behavioural exercises and were not the focus of this review.

### Search strategy and selection process

A comprehensive search of studies was conducted from inception until 10 February 2022 using the electronic databases: MEDLINE, Embase via Ovid, CINAHL, Web of Science and SpeechBITE. The Medical Subject Heading terms: “*Deglutition, Deglutition Disorders, Pharynx* OR *Pharyngeal Muscles”* were combined with “*Stroke* OR *Cerebrovascular Disorders”;* and “*Exercise, Exercise Therapy, Neurological Rehabilitation, Stroke Rehabilitation* OR *Rehabilitation”* along with free key word searches of specific swallowing exercises. The search strategy was developed in conjunction with a university librarian using candidate search terms from two relevant studies. See Online Appendices 1–3 for full search strategies for each database. The reference lists of included studies were hand searched to identify further studies.

Covidence software was used to remove duplicates and double checked by the lead author [20]. Two reviewers independently screened titles, abstracts, and eligible full text articles against inclusion criteria using Covidence software. Conflicts were resolved through discussion with a third reviewer. Abstracts or full text studies which were not in English were translated by bilingual speakers. All members of the research term agreed on the final studies included for review.

### Data collection

Data was extracted from included studies by the lead author using an Excel form and checked by a second reviewer. The following data was extracted:

- Study author, year, and source of publication.
- Participant demographics (sample size, age, sex, inclusion/exclusion criteria, length of time since stroke) and participant diagnoses (cause of dysphagia, stroke type and severity).
- Setting and study design (including study aims and intervention groups).
- Dosage of swallowing exercises (type of exercises, any reported intensity, frequency of sets/sessions and duration).
- All outcomes pre and post intervention (excluding follow-up timepoints).

### Risk of bias assessment

Included studies were assessed for quality by two independent reviewers using the American Speech–Language–Hearing Association (ASHA) Levels of Evidence framework on an online spreadsheet tool [21, 22]. The framework involved rating studies against eight quality markers: blinding of assessors, random sampling/allocation, group/participant comparability, treatment fidelity, validity and reliability of an outcome measure, whether significance was reported, precision of effect size and/or confidence interval and analysis by intent-to-treat. Each quality marker contributed to one point in an overall quality score. A quality score of 7–8 was considered high quality, 5–6 good quality and ≤ 4 low quality [21]. Conflicts were resolved through discussion with a third reviewer.

### Summary measures

Information about participants, swallowing exercises, reported dosages and outcome measures was collated into two summary tables. Tables were organised alphabetically by exercise type to allow comparison of swallowing exercise dosages. When summary data was missing or in a different form, means and standard deviations were estimated using Hozo et al. or Wan et al.’s methods [23, 24]. Effect sizes (Hedges’ *g*) were calculated for pre–post changes in continuous data for swallowing intervention groups using an online effect size calculator [25]. Hedges’ *g* effect sizes can be interpreted as 0.2 = small effect, 0.5 = medium effect, 0.8 = large effect [26]. Meta-analysis could not be conducted due to heterogeneity of study designs, interventions, dosages, and outcome measures, and was not necessary to address study objectives.

## Results

**Fig. 1:**
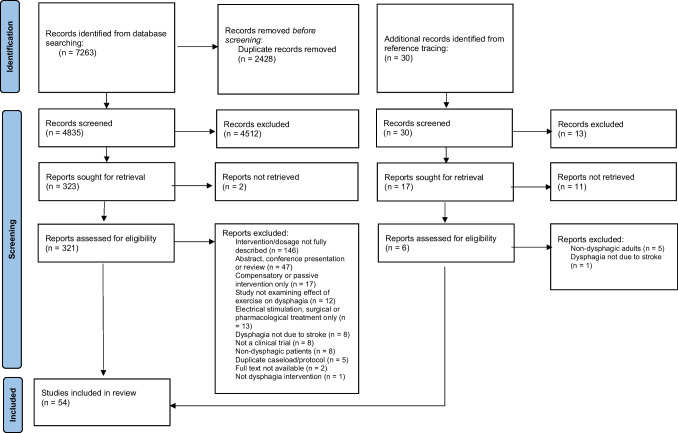
PRISMA flow diagram

The initial search resulted in 7263 studies. After duplicates were removed, 4835 studies were screened for inclusion. Of these, 54 studies passed full text review (Fig. 1).

### Participant characteristics

Within the 54 included studies were a total of 1501 participants with an average age of 65.8 years. Study sample sizes ranged from one [27–30] to 90 [31] participants. Seven studies had mixed caseloads, including patients with dysphagia due to cancer, brain injury, degenerative and/or cardiac conditions [32–38]. The remaining studies included participants with stroke-related dysphagia only, with a variety of stroke types, locations, and severities. Eleven studies included ischemic stroke only [30, 31, 37, 39–46] and sixteen studies included both ischemic and haemorrhagic stroke [47–55, 57–60, 71, 72, 81]. Nine studies examined supratentorial strokes [41, 42, 46–48, 57, 58, 61, 62], six studies examined infratentorial strokes [27–30, 56, 63] and twelve studies included both supratentorial and infratentorial strokes [40, 45, 51, 59, 60, 64–69, 81]. Seven studies did not report stroke type or location. Only two studies reported stroke severity scores [30, 37].

The length of time between stroke and commencement of intervention was reported in 50 studies. Twenty-five studies were conducted within 6 months after stroke, 16 conducted more than 6 months after stroke and nine studies included participants across a range of time periods post-stroke. See Tables 1 and 2 for sample sizes, participant ages, and length of time between stroke and onset of intervention as reported in each study.

**Table 1:** Single swallowing interventions in included studies (including participants, exercise descriptions and dosages, outcome measures and pre–post Hedges *g* effect sizes in exercise-based intervention group) grouped by exercise type

Study	Number of participants (mean age in years ± SD)	Mean time post CVA ± SD	Description of exercise	Intensity	Frequency (per day/week)	Duration	Outcome measures	Effect sizes (if *p* < 0.05)
Cervical isometric strength training								
Ploumis et al. (2018) [70]	T: 70 (52 ± 15) Exp^a^: 37 Con: 33	2.5 ± 1.1 d	Contract neck muscles forwards–backwards–sidewards against resistance	4 reps in all 4 directions for 10 min	3x/day	12 weeks	Change of sagittal and coronal C2–C7 Cobb angle VFSS score	NR
Chin tuck against resistance								
Gao and Zhang (2017) [31]	T: 90 CTAR^a^: 30 (70.88 ± 6.6) Shaker: 30 (71.12 ± 7.07) Con: 30 (71.14 ± 6.41)	CTAR: 12.95 ± 1.60 d Shaker: 13 ± 1.41 d Con: 12.15 ± 1.35 d	Chin tuck against an inflatable rubber ball as far as possible while seated	30 reps	3x/day, 7x/week	6 weeks	PAS	*g* = 1.33
Kim and Park (2019) [71]	CTAR^a^: 12 (63.5 ± 5.5) Con: 13 (65.2 ± 6.2)	< 6 mos	PhagiaFlex-HF device fixed to the desk and height adjusted so device was under the chin. Chin down exercises performed against device	Isometric: 10 s hold × 3 Isotonic: 30 reps	5x/wk	6 wks	PAS FOIS	*g* = 1.99 *g* = 1.60
Park et al. (2018) [52]	CTAR^a^: 11 (62.16 ± 17.27) Con: 11 (58.43 ± 12.51)	Exp: 37.24 ± 8.54 wks Con: 32.14 ± 14.38	Isometric and isokinetic chin tucks against CTAR device as strongly as possible in sitting position	Isometric: 60 s hold × 3 Isokinetic: 30 reps	5x/wk	4 wks	FDS PAS	*g* = 1.02 *g* = 1.69
Park et al. (2019) [72]	CTAR^a^: 19 (60.95 ± 11.19) Shaker: 18 (59.45 ± 9.34)	CTAR: 3.6 ± 1.19 Shaker: 3.85 ± 1.18	LES 100 CTAR device was used with a resistance bar placed beneath the chin. 1-RM was determined. A tablet PC was used to display targets	70% of 1-RM. Isometric: 60 s chin tuck × 3 Isotonic: 30 consecutive reps	5x/day	4 wks	VDS oral phase VDS pharyngeal phase VDS total PAS FOIS	*g* = 0.92 *g* = 2.16 *g* = 2.34 *g* = 1.54 *g* = 1.16
Effortful swallowing								
Cho et al. (2017) [73]	9 (age NR)	NR	Press tongue firmly against the palate while swallowing as hard as possible	30 reps	5x/wk	4 wks	VDS oral phase VDS pharyngeal phase	*g* = 0.74 *g* = 0.32
Park et al. (2019) [62]	Exp^a^: 12 (66.5 ± 9.5) Con: 12 (64.8 ± 11.2)	Exp: 24.39 ± 8.65 wks Con: 25.74 ± 6.27 wks	Push tongue firmly onto palate while squeezing neck muscles and swallow as forcefully as possible	10 reps	3x/day, 5x/wk	4 wks	Ant. tongue strength Post. tongue strength VDS	*g* = 1.51 *g* = 1.23 *g* = 1.32
Wei et al. (2017) [63]	Con^a^: 15 (57.9 ± 9.3) Exp: 15 (57.7 ± 8.8)	Con:4.5 ± 2.3 mos Exp:4.3 ± 2.6 mos	Swallow as hard as possible using mouth muscles without recruiting abdominal/stomach muscles	10 reps per day	2x/day, 5x/wk	3 wks (or if no longer tube fed)	Motor evoked potentials UES displacement Max UES opening FOIS	NR
Expiratory muscle strength training (EMST)/Respiratory training								
Arnold et al. (2020) [45]	Exp^a^: 10 (70.5 ± NR) Con: 10 (66.1 ± NR)	NR	Forcefully inhale and exhale through Breather device using diaphragmatic breathing technique with nose clip in place	5 min × 3 sets at highest tolerated settings on device	3x/day, 7x/wk	4 wks	PEFR PAS FOIS VAS MASA	*g* = 3.04 *g* = 2.19 *g* = 1.63 *g* = 2.15 *g* = 2.32
Eom et al. (2015) [64]	Exp^a^: 13 (69.2 ± 4.1) Con: 13 (70.2 ± 3.6)	< 3 mos	Blow strongly and rapidly into EMST device until pressure release valve opens (at > 70% of MEP)	5 breaths (< 1 min break between sessions)	5x/day, 5x/wk	4 wks	VDS PAS	*g* = 1.68 *g* = 1.49
Guillen-Sola et al. (2017) [37]	IEMT^a^: 21 (67.9 ± 10.6) NMES: 20 (70.3 ± 8.4) Con: 21 (68.9 ± 7)	IEMT:10.8 ± 8.7 d NMES: 11 ± 5.5 d Con: 9.3 ± 5.1 d	Respirations at 30% of max inspiratory and expiratory pressures and increased by 10cmH_2_O each week. 1 min of recovery breathing off the device	5 sets of 10 reps	2x/day, 5x/wk	3 wks	PAS FOIS DOSS Max. inspiratory pressure MEP	NR
Hegland et al. (2016) [65]	14 (64.5 ± 7.4)	11.9 ± 7.9 mos	With noseclips on, exhale quickly and forcefully into EMST device until valve opens (at 60% of MEP)	5 reps	5x/day, 5x/wk	5 wks	MEP PEPR vol. cough PEPR cough reflex VA vol. cough VA cough reflex MBSImp PAS	*g* = 0.85 n/s *g* = 1.41 n/s *g* = 0.67 *g* = 1.55 NR
Liaw et al. (2020) [55]	Exp^a^: 11 (65.4 ± 11.54) Con: 10 (60.44 ± 10.65)	≥ 6 mos	Inspiratory muscle training (IMT): inhale deep and forceful breaths sufficient for opening the valve. Expiratory muscle training (EMT): blow fast and forcefully to open the valve	IMT: 30–60% of max pressure EMT: 15–75% of MEP × 5 reps for 5 sets each	1–2x/day (1–2 min rest), 5x/wk	6 wks	Change in max. inspiratory pressure Change in MEP FVC FEV1 FOIS	*g* = 1.33 *g* = 1.10 n/s n/s *g* = 1.11
Moon et al. (2017) [50]	Exp^a^: 9 (63 ± 5.8) Con: 9 (63.1 ± 5.2)	Exp: 21.4 ± 5.1 d Con: 21.1 ± 4 d	Take a deep breath and bite the EMST mouthpiece. Close nostrils and blow fast and strong into device (set at 70% of MEP)	7 reps	5x/wk	4 wks	FDS PAS Vallecular residue Pyriform sinus residue	*g* = 1.70 *g* = 1.79 *g* = 2.05 *g* = 0.93
Park et al. (2016) [67]	Exp^a^: 14 (64.3 ± 10.7) Con: 13 (65.8 ± 11.3)	Exp:27.4 ± 6.3 wks Con:26.6 ± 6.8 wks	After max inhalation, blow strong and fast into EMST device between lips until pressure release valve opens (at 70% MEP)	5 reps (< 1 min break after each session)	5x/day, 5x/wk	4 wks	Suprahyoid muscle activity PAS (liquids) PAS (semisolids) FOIS	*g* = 1.14 *g* = 1.93 *g* = 1.02 *g* = 1.47
Game-based biofeedback								
Stepp et al. (2011) [28]	1 (18)	6 yrs	Swallow at target strength/length (33%, 66% and 100% of max swallow strength for 2.8, 3.5 or 4.7 s) to “eat” the 7 swallow targets (depicted as fish on sEMG machine)	7 reps for 10 sets of 2 min (1–2 min breaks and > 5 min between sets 5–6)	3x/wk	3 wks	Number of targets per session Neck intermuscular beta coherence	NR
Jaw opening exercise								
Choi et al. (2020) [58]	Jaw opening^a^: 11 (63.5 ± 7.7) Head lift: 10 (61.2 ± 9.7)	Jaw opening: 12.1 ± 2.2wks Head lift: 13.4 ± 2.2wks	Hold jaw open against resistance bar (isometric). Open jaw against resistance bar (isotonic)	Isometric: 10 s hold × 3 reps Isotonic: 3 sets of 30 reps	5x/wk	6 wks	Muscle thickness: Digastric Mylohyoid Hyoid movement: Anterior Superior BRPES	*g* = 0.62 *g* = 1.06 *g* = 1.1 *g* = 0.89 NR
Koyama et al. (2017) [66]	Exp^a^: 6 (66.0 ± 9.3) Con: 6 (71.8 ± 7.6)	Exp: 6.7 ± 2.1 mos Con: 9.2 ± 4 mos	Press tongue against hard palate. Open mouth against resistance (trainer’s hand pushing chin up). Muscle contraction measured with surface electrodes	80% MVC 6 s hold × 5 reps	4x/day, 5x/wk	6 wks	Distance between mental spine and hyoid bone Hyoid displacement: Superior Anterior	*g* = 0.41 n/s *g* = 1.26
Oh et al. (2017) [74]	3 (age NR)	< 12mos	Open mouth against external resistance (38 cm circumference ball)	Isometric: hold for 60 s, Isotonic: 30 reps	5x/wk	4 wks	PAS (liquids)	*g* = 2.04^b^
Park et al. (2020) [54]	Exp^a^: 20 (62.1 ± 10.1) Con: 20 (61.8 ± 12.1)	< 6mos	Resistive jaw opening device affixed to the sternum and resistive portion placed below chin. Depress the resistive jaw opening device	Isometric: hold for 30 s × 3 reps Isotonic: 2–3 s × 10 reps (30 s rest)	3x/day, 5x/wk	4 wks	Hyoid movement: Anterior Superior PAS (semisolids) PAS (liquids) FOIS	*g* = 0.9 *g* = 0.7 *g* = 0.6 *g* = 0.6 *g* = 1.1
Wada et al. (2012) [35]	8 (70.5 ± 11.3)	Chronic	Open jaw to max extent	10 s hold × 5 reps (10 s rest between reps)	2x/day, daily	4 wks	Movement of hyoid: Upward Forward UES opening width Time for pharynx passage	*g* = 0.99 n/s *g* = 0.36 *g* = 0.90
Lip muscle training								
Hagg and Anniko (2008) [75]	30 (70 ± 9.75)	1 mos (2 days–10 yrs)	Hold an oral screen predentally between closed lips as screen is gradually pulled away	5–10 s hold × 3 reps	3x/day, 7x/wk	At least 5 wks	Lip force meter Swallowing capacity test	*g* = 1.40 *g* = 1.79
Hagglund et al. (2020) [59]	Exp^a^: 18 (75, range: 56–90) Con: 14 (75, range: 60–85)	NR	Hold oral device (Muppy) predentally behind closed lips against a gradually increasing horizontal pulling force	5–10 s hold × 3 reps	3x/day, daily	5 wks	TWST Lip force PAS	NR NR NR
Park et al. (2018) [76]	10 (age NR)	≤ 6 mos	Press IOPI bulb between lips	70% of 1–RM × 30 reps/wk	5x/wk	4 wks	Lip strength Lip closure on VFSS	*g* = 0.98 *g* = 1.05
Masako maneuver								
Kumaresan et al. (2018) [77]	30 (age NR)	NR	Protrude tongue and gently bite down on the anterior part of the tongue while swallowing saliva	10 reps × 3	3x/day	2 wks	EAT-10	*g* = 9.86
Mendelsohn maneuver								
Bogaardt et al. (2009) [36]	11 (61.1 ± 7.6)	30.6 ± 42.4mos	Modified maneuver: prolong laryngeal excursion for 8–10 s with sEMG feedback	Instructed to practise without sEMG 2–3x/day (40–60 reps)	1x/wk (or 1x/fort-night)	6.4 sessions and 76.1 days (mean)	FOIS	*g* = 1.46
McCullough and Kim (2013) [78]	Crossover group: 18 (70.2 ± 11.5; range: 42–88)	9.5 ± 4; range: 6–22mos	Swallow “long and strong” with a squeeze at the peak of the swallow for 3–4 s with sEMG feedback	Target set at 5 μV above mean 30–40 swallows, 45–60 min	2x/day (2–3 h breaks)	2 wks	Max hyoid: Anterior excursion Elevation UES opening width Duration of: Hyoid elevation UES opening DOSS	n/s n/s n/s n/s n/s n/s
Wei et al. (2017) [63]	Con^a^: 15 (57.9 ± 9.3) Exp: 15 (57.7 ± 8.8)	Con: 4.5 ± 2.3 mos Exp: 4.3 ± 2.6 mos	Swallow the bolus by pressing tongue against hard palate and squeezing the throat muscles. Maintain swallow for 2 s	10 reps	2x/day, 5x/wk	3 wks (or if no longer tube fed)	Motor evoked potentials UES displacement Max UES opening FOIS	NR
Proprioceptive Neuromuscular Facilitation (PNF)-based short neck flexion exercises								
Kim et al. (2015) [79]	Exp^a^: 13 (63.2 ± 10.2) Con: 13 (63.6 ± 8.1)	Exp:15.6 ± 2.9 mos Con:16.15 ± 3.1 mos	While supine with head/neck off the bed, look at target object 15° diagonally to the right, while the tester moves the participant’s neck in the opposite direction. Repeat in the opposite direction	30 min	3x/wk	6 wks	New VFSS scale ASHA NOMS	*g* = 1.97 *g* = 0.67
Shaker head lift								
Cho et al. (2017) [73]	9 (age NR)	NR	Lift the head while in lying position (isometric and isokinetic)	Isometric: 60 s hold Isokinetic: 30 reps	5x/wk	4 wks	VDS oral phase VDS pharyngeal phase	*g* = 0.74 *g* = 0.32
Choi et al. (2017) [47]	Exp^a^: 16 (60.81 ± 10.85) Con: 15 (60.4 ± 10.5)	Exp:3.44 ± 1.15mos Con: 4.13 ± 0.99mos	Head lift high enough to observe toes in supine position (isometric and isokinetic)	Isometric: 60 s hold × 3 reps (60 s rest) Isokinetic: 30 reps	5x/wk	4 wks	PAS FOIS	*g* = 2.06 *g* = 1.57
Choi et al. (2020) [58]	Head lift^a^: 10 (61.2 ± 9.7) Jaw opening: 11 (63.5 ± 7.7)	Head lift: 13.4 ± 2.2wks Jaw opening: 12.1 ± 2.2wks	Lift head and hold (isometric). Lift head and lower (isotonic)	Isometric: 10 s hold × 3 reps Isotonic: 3 sets of 30 reps	5x/wk	6 wks	Muscle thickness: Digastric Mylohyoid Hyoid movement: Anterior Superior BRPES	*g* = 0.91 *g* = 1.28 *g* = 1.29 *g* = 0.91 NR
Gao and Zhang (2017) [31]	Shaker^a^: 30 (71.1 ± 7.07) CTAR:30 (70.88 ± 6.6) Con:30 (71.14 ± 6.41)	Shaker: 13 ± 1.41 d CTAR:12.95 ± 1.6 d Con:12.15 ± 1.35 d	Raise head and neck to look at feet from supine position (isokinetic only)	30 reps	3x/day, 7x/wk	6 wks	PAS	*g* = 1.33
Kim et al. (2015) [79]	Con^a^: 13 (63.6 ± 8.1) Exp: 13 (63.2 ± 10.2)	Con:16.15 ± 3.1 mos Exp:15.6 ± 2.9 mos	Lie on bed and raise head without moving shoulders to look at feet and hold (isometric and isokinetic)	Isometric: 60 s × 3 (60 s rest) Isokinetic: 30 reps 30 min	3x/wk	6 wks	New VFSS scale ASHA NOMS	*g* = 1.48 *g* = 2.33
Logemann et al. (2009) [32]	Exp^a^: 8 (63.1 ± 22.8) Con: 11 (70.9 ± 9.5)	> 3mos	Isometric head lifts in supine position (with rest between lifts) followed by consecutive isokinetic reps	Isometric: 60 s × 3 Isokinetic: 30 reps	Practice 3x/day, 2x/wk	6 wks	Hyoid movement: Anterior Superior	n/s n/s
							Laryngeal movement: Anterior Superior Max UES opening	n/s n/s n/s
Mepani et al. (2009) [34]	Exp^a^: 5 (64 ± 22.8) Con: 6 (70.5 ± 9.5)	> 3 mos	Raise head high and forward enough to observe toes while in supine position. Isometric head lifts with rest period followed by consecutive head lifts at constant velocity without rest (isokinetic)	Isometric: 60 s hold × 3 (60 s rest) Isokinetic: 30 reps. 45 min	2x/wk	6 wks	Thyrohyoid muscle shortening	*g* = 1.08
Park et al. (2017) [53]	Exp^a^: 13 (59.26 ± 11.94) Con: 14 (61.59 ± 13.61)	Exp: 21.29 ± 8.92 wks Con: 19.2 ± 5.65 wks	Raise head to look at toes and hold (isometric) without lifting shoulders in supine position (rest period between lifts) followed by × 30 isokinetic consecutive reps	Isometric: 60 s hold × 3 (60 s rest) Isokinetic: 30 reps	5x/wk	4 wks	PAS (liquids) PAS (semisolids) Hyoid displacement: Horizontal Vertical Larynx displacement: Horizontal Vertical	*g* = 1.78 *g* = 0.97 *g* = 1.01 *g* = 1.25 *g* = 0.48 *g* = 0.57
Park et al. (2019) [72]	Shaker^a^: 18 (59.45 ± 9.34) CTAR: 19 (60.95 ± 11.19)	Shaker: 3.85 ± 1.18 CTAR: 3.6 ± 1.19	Raise head to look at toes and hold (isometric) without lifting shoulders in supine position, followed by isokinetic consecutive reps	Isometric: 60 s hold × 3 Isotonic: 30 consecutive reps	5x/day	4 wks	VDS oral phase VDS pharyngeal phase VDS total PAS FOIS	*g* = 0.98 *g* = 1.71 *g* = 1.87 *g* = 0.95 *g* = 0.74
Shaker et al. (2002) [38]	27 (73.37 ± 6.21)	8.63 ± 18.54 mos	Lie flat and perform sustained head raisings (isometric) with rest periods, followed by consecutive isokinetic head raisings (high and forward enough to observe toes)	Isometric: 60 s hold × 3 (60 s rest) Isokinetic: 30 reps	3x/day	6 wks	UES opening Laryngeal excursion Anterior Superior Hyoid excursion FOAMS	*g* = 3.48 *g* = 1.91 n/s n/s *g* = 6.66
Swallowing with kinesiology taping								
Jung et al. (2020) [60]	Exp^a^: 13 (71.3 ± 6.5) Con: 14 (70.5 ± 8.2)	Exp: 16.2 ± 5.2 wks Con: 15.1 ± 6.4 wks	Voluntary swallow with kinesiology tape attached to the hyolaryngeal complex, pulled downward with approx. 70% tension and attached to the sternum and clavicle bilaterally	5 sets of 10 swallows against kinesiology tape tension	10x/day, daily	4 wks	Muscle thickness: Tongue Mylohyoid Anterior belly of digastric	*g* = 0.55 *g* = 1.24 *g* = 0.83
Supraglottic swallow								
Wei et al. (2017) [63]	Con^a^: 15 (57.9 ± 9.3) Exp: 15 (57.7 ± 8.8)	Con:4.5 ± 2.3 mos Exp:4.3 ± 2.6 mos	Hold breath before and during swallow, and cough/clear throat after swallow before breathing	10 reps	2x/day, 5x/wk	3 wks (or if no longer tube fed)	Motor evoked potentials UES displacement Max UES opening FOIS	NR
Tongue strengthening exercises								
Cho et al. (2017) [73]	9	NR	Press tongue strongly against hard palate	30 reps	5x/wk	4 wks	VDS oral phase VDS pharyngeal phase	*g* = 0.74 *g* = 0.32
Juan et al. (2013) [27]	1 (56)	27 mos	Press tongue against bulb placed between hard palate and either anterior or posterior tongue	60% of 1-RM for 1st wk; 80% of 1-RM onwards × 10 reps	Therapy: 3x/day, 3x/wk	8 wks therapy	Lingual pressures Ant Post Lingual volume SWAL–QOL	N/A
Kim et al. (2017) [61]	Exp^a^: 18 (62.17 ± 11.01) Con: 17 (59.29 ± 10.19)	Exp: 4.94 ± 5.52 mos Con: 5.29 ± 5.62 mos	Press tongue strongly against palate for anterior and posterior tongue regions in a random sequence	30 reps	5x/wk	4 wks	Ant. tongue strength Post. tongue strength VDS oral phase VDS pharyngeal phase PAS	*g* = 0.89 *g* = 1.41 *g* = 1.06 *g* = 0.91 *g* = 2.26
Moon et al. (2018) [51]	Exp^a^: 8 (62 ± 4.17) Con: 8 (63.50 ± 6.05)	Exp: 56 ± 17.35 d Con: 59.88 ± 20.04 d	Ex_1_: Press tongue tip on bulb at posterior alveolar arch and press middle portion of tongue on bulb at middle of the hard palate Ex_2_: generate precise pressures (± 10 kPa of target)	Ex_1_: 6 reps × 5 Ex_2_: Targets at 50, 75 and 100% of max pressure 30 min	5x/wk	8 wks	Ant. MIPs Post. MIPs MASA SWAL–QOL	*g* = 3.17 *g* = 4.50 *g* = 5.73 *g* = 3.40
Park et al. (2015) [49]	Exp^a^: 15 (67.3 ± 10.6) Con: 14 (65.8 ± 11.5)	Exp: 25.37 ± 7.43 wks Con: 26.38 ± 6.81 wks	Press IOPI bulb toward hard palate with tongue as hard as possible in anterior and posterior positions	2 s hold at 80% of 1-RM × 10 reps	5x/day (min. 30 s rest), 5x/wk	6 wks	Ant. MIPs Post. MIPs VDS	*g* = 0.26 *g* = 0.50 *g* = 0.36
Robbins et al. (2007) [68]	10 (69.7 ± 13.66)	1 mos (*n* = 6), 5 to > 48 mos (*n* = 4)	Compress IOPI bulb between tongue and hard palate for anterior and posterior tongue	60% of 1-RM for 1st wk and 80% of 1-RM onwards × 10 reps	3x/day, 3x/wk	8 wks	Change in MIPs (IOPI) Swallowing pressure Oropharyngeal residue PAS Durational measures MRI SWAL–QOL Dietary questionnaires	NR
Steele et al. (2016) [69]	14 (71 ± 13.43)	70.29 ± 42.21 d	*Strength training:* Ex_1_: post. MIPs, regular and effortful saliva swallows Ex_2_: post. MIPs with slow-release trials Ex_3-4_: effortful and regular saliva swallows with slow-release trials Ex_5_: nectar-thick liquid swallows with slow-release *Accuracy training:* Ex_1_: ant. and post. MIPs Ex_2-3_: ant. and post. tongue MIP trials Ex_4-5_: ant. and post. tongue target accuracy trials	*Strength:* Ex_1_: 5 reps each Ex_2_: 20 reps Ex_3-4_: 10 reps each Ex_5_: 5 reps *Accuracy:* Ex_1_: 5 reps Ex_2-3_: 10 reps Ex_4-5_: 15 reps each (25–85% of MIP)	2–3x/wk	8–12 wks	Post. MIPs Stage transition duration (from VFSS) PAS Normalised Residue Ratio Scale	NR n/s n/s NR NR
Yeates et al. (2008) [29]	1 (72)	7 mos	Ant. and post. tongue-to-palate presses (half isometric strength, half accuracy) *Strength*: press bulb to roof of mouth as hard as possible. 1 set of tongue presses in ant. position coupled with a swallow *Accuracy:* generate precise pressures in ant. or post. tongue positions	6 reps × 10 sets in total *Strength:* at max pressure *Accuracy:* 50, 75, 100% of MEP	10 sets per day, 2–3x/wk	24 sessions	Ant. and post. tongue bulb pressure (average, accuracy, accuracy relative to strength) Stage transition duration (from VFSS)	N/A

*1-RM* 1 repetition maximum, ant. anterior, *ASHA NOMS* American Speech–Language Hearing Association’s National Outcomes Measurement System, *BRPES* Borg rating of perceived exertion, *C2–C7* cervical vertebrae 2–7, *Con* control group, *CTAR* chin tuck against resistance, *CVA* cerebrovascular accident**,** *d* days, *DOSS* Dysphagia Outcome and Swallow Scale, *EAT-10* Eating Assessment Tool-10**,** *EMST* expiratory muscle strength training, *Ex*_*1,2*_ exercise 1, 2 etc., *Exp* experimental group, *FDS* Functional Dysphagia Scale, *FEV1* forced expiratory volume per second, *FOAMS* Functional Outcome Assessment of Swallowing, *FOIS* Functional Oral Intake Scale**,** *FVC* forced vital capacity, *g* Hedges’ *g*, *h* hour/s, *H*_*2*_*O* water, *IEMT* Inspiratory/Expiratory Muscle Training, *IOPI* Iowa Oral Performance Instrument, *kPa* kilopascals, *LEDT* laryngeal elevation delay time, *MASA* Mann Assessment of Swallowing Ability, *max* maximum, *MBSImp* Modified Barium Swallow Impairment Profile, *min* minute/s, *min.* minimum, *MEP* maximum expiratory pressure, *MIP* maximum isometric pressure, *mos* months, *MRI* magnetic resonance imaging, *MVC* maximum voluntary contraction, *NMES* neuromuscular electrical stimulation, *NR* not reported or not calculable, *n/s* not significant, *PAS* Penetration–Aspiration Scale, *PEFR* peak expiratory flow rate**,** *post.* posterior, *reps* repetitions, *RSST* Repetitive Saliva Swallowing Test, *s* seconds, *sEMG* surface electromyography, *SD* standard deviation, *SWAL–QOL* Swallowing Quality-of-Life questionnaire, *T* total, *TWST* Timed Water Swallow Test, *μV* microvolts, *UES* upper oesophageal sphincter, *VA* volume acceleration, *VAS* visual analogue scale, *VDS* Videofluoroscopic Dysphagia Scale, *vol* voluntary, *VFSS* Videofluoroscopic Swallow Study, *vs* versus, *wk/s* week/s, *WST* Water Swallowing Test (WST), *x/*times per, *yrs* years

^a^Intervention, dosage and effect sizes reported for the first listed group (with studies containing > 1 group)

^b^Not reported whether results were significant or not

**Table 2:** Combined intervention programs in included studies (including participants, exercise descriptions and dosages, outcome measures and pre–post Hedges *g* effect sizes in exercise-based intervention group)

Study	Number of participants (mean age in yrs ± SD)	Mean time post CVA ± SD	Summary of exercises	Intensity	Frequency (per day/wk)	Duration	Outcome measures	Effect sizes (if *p* < 0.05)
El-Tamawy et al. (2015) [39]	Exp^a^: 15 (61.53 ± 7.26) Con: 15 (61.33 ± 6.57)	NR	Ex_1_: Tongue exercises, Ex_2_: Jaw exercises, Ex_3_: Swallow boluses, Ex_4_: Vibrate laryngeal musculature downward, Ex_5_: Push head and neck in flexion, extension and lateral flexion vs resistance	70–75 min 10 reps of each exercise	3x/wk	6 wks	Oral transit time	*g* = 1.47
							Hyoid and laryngeal elevation	NR
							Oesophageal sphincter opening	NR
							Aspiration/penetration	NR
Hamzic et al. (2021) [30]	1 (57)	NR	Ex_1_: Ice stimulation of anterior faucial arches Ex_2_: Chin tuck against resistance Ex_3_: Jaw opening exercise	Ex_1_: 5 times each side Ex_2-3_: 10 s hold × 5 reps for 5 sets, 10 s break between reps	3x/day, 5x/wk sessions (daily home practice)	7 wks	PAS	NR
							Yale Pharyngeal Residue Severity Rating Scale	
							Valleculae	NR
							Pyriform sinuses	NR
							FOIS	NR
Jiao et al. (2022) [46]	Con^a^: 32 (57.3 ± 9.1) Exp: 29 (60 ± 10.6)	NR	Basic rehabilitation training: Ex_1_: Suck training, Ex_2_: Tongue muscle training, Ex_3_: Pharyngeal cold stimulation, Ex_4_: Pronunication of “a”, “yi”, “wu”, Ex_5_: Facial muscle training, Ex_6_: Neck muscle training, Ex_7_: Food intake training	20 reps of each exercise	2x/day, 5x/wk	2 wks	WST	NR
Kim (2010) [40]	8 (58.75 ± 6.40)	NR	Music-enhanced swallowing treatment:	15 min	3x/wk	4 wks	Frenchay including dysphagia subtests:	
			Ex_1_ (2 min): pitch glides Ex_2_ (3 min): sing song	Ex_1-2_: 1 rep each			Cough	n/s
							Swallow	n/s
							Drool	*g* = 0.81
			Ex_3_ (4 min): 2-step and 3-step breathing (with 2–3 s breath hold after inhalation) Ex_4_ (3 min): hum notes using vowel sounds	Ex_3-4_: 10 reps each				
Krajczy et al. (2019) [80]	Exp^a^: 30 (62.1 ± 3.3) Con: 30 (64.1 ± 1.5)	Acute stage	Ex_1_: Lip exercises. Ex_2_: Tongue ROM and resistance exercises.	60 min Ex_1-3_: × 10 reps each		15 days	Swallow reflex	NC
							Assessment of coughing	NC
			Ex_3_: Cheek exercises Ex_4_: Breathing exercises	Ex_4_: Duration as per patient			Timed swallow test	*g* = 0.74
							Average number of swallows	*g* = 0.77
			Ex_5_: Swallow after thermal–tactile stimulation	Ex_5_: × 5 swallows			SpO_2_ (after test)	*g* = 0.27
Li et al. (2019) [56]	A (swallow Tx) ^a^: 15 (57 ± 7)	A: 2.2 ± 1.1	Ex_1_: Active lip, tongue and resistance exercises Ex_2_: Rapidly start swallowing	Ex_1_: 5 s hold × 5 reps	1x/day, 5x/wk	6 wks (or after removal of tube)	VFSS dysphagia score	*g* = 0.75^b^
							Oral	*g* = 0.61^b^
							Pharyngeal	*g* = 0.72^b^
							Aspiration	NC
	B: 15 (57 ± 8) C1: 15 (57 ± 8) C2: 15 (60 ± 6)	B: 2.5 ± 1.4 C1: 2.5 ± 1.8 C2: 2.9 ± 2.6 C3: 2.4 ± 1.1 D: 2.5 ± 1.6	Ex_3_: Respiration training	Ex_2_: × 10–15 reps Ex_3_: × 5 reps each as long as tolerated			Evaluation of swallowing function (Fujishima Ichiro standard)	NC
	C3: 15 (58 ± 8) D: 15 (56 ± 8)		Ex_4_: Masticatory muscle training	Ex_4_: 5 s hold × 5 reps			Extubation rate	NC
Malandraki et al. (2016) [33]	10 (64.6 ± 14.5)	8.89 ± 9.70 mos	Ex_1_: Regimen A and B practised on alternating days. Regimen A: lingual strengthening (IOPI), tongue hold with mirror, effortful swallow with VAS. Regimen B: effortful swallow and Mendelsohn with VAS, head lift with timer Ex_2_: Daily targeted swallow with materials identified from FEES (and consistencies changed based on performance)	Ex_1_: Re-evaluated and increased either target goals, reps or duration every 1–2 wks Ex_2_: 20 swallows × 3	2x/wk, Daily home practice 45 min/day	4 wks	PAS	NC
							Lingual pressures	
							Anterior	*g* = 0.96^b^
							Posterior	*g* = 0.54^b^
							EAT-10	*g* = 1.41
							ASHA NOMS	NC
Momosaki et al. (2014) [41]	4 (66.25 ± 9.0)	29.25 ± 5.2 mos	Ex_1_: Jaw, cheek and tongue exercises	20 min Ex_1_: 10–20 reps each	2x/day	6 consecutive days	PAS	*g* = 1.98^b^
							LEDT	*g* = 1.44^b^
							FOIS	*g* = 0.46^b^
			Ex_2_: Push tongue up against resistance	Ex_2_: 10 s hold × 5 reps (3 s rest)			MASA	*g* = 0.29^b^
							RSST	*g* = 0.45^b^
			Ex_3_: Isokinetic Shaker	Ex_3_: 10 reps × 3 (30 s rest)				
Momosaki et al. (2015) [42]	8 (65 ± 2.7)	33.5 ± 4.2 mos	(as per Momosaki et al. [41])	(as per Momosaki et al. [41])	(as per Momosaki et al. [41])	(as per Momosaki et al. [41])	MASA	*g* = 0.95
							PAS	NR
							LEDT	*g* = 0.41
							SWAL–QOL	*g* = 0.84
							FOIS	NR
Moon et al. (2019) [81]	Exp^a^: 8 (54.13 ± 5.41) Con^a^: 8 (55.38 ± 14.88)	Exp: 22.75 ± 9.21, Con: 21.00 ± 9.02	Orofacial exercise program: jaw, lip, tongue and cheek exercises	20 min × 5 reps each	3x/day, 3x/wk	4 wks	FDS	*g* = 2.08
							PAS	*g* = 1.97
							VASS	NC
Oh et al. (2013) [48]	Exp^a^: 7 (53.71 ± 12.46)	Exp:43 ± 27.9 mos	Stomatognathic therapy: Ex_1_: pull chin back (in supine)	Ex_1, 3_: 10 s hold × 10 reps	3x/wk	4 wks	MASA	*g* = 0.81
							Mouth opening range	*g* = 0.48
							Craniomandibular index	*g* = 0.96
	Con: 7 (56.14 ± 12.31)	Con: 13.57 ± 16.53 mos	Ex_2_: pull chin back (standing against wall) Ex_3_: head and shoulders pressed against wall while maintaining chin tuck and straight back, arms at 45° shoulder abduction and elbows fully extended Ex_4_: neck and jaw exercises	Ex_2_: 2 min hold × 5 reps Ex_4_: 10 s hold × 10 reps × 2 sets			Neck mobility:	
							Flexion	*g* = 0.80
							Extension	*g* = 0.40
							Rotation	*g* = 0.61
							Lateral flexion	*g* = 0.73
Xing et al. (2019) [82]	Con^a^: 50 (67 ± 7.2) Exp: 50 (66.9 ± 7.3)	Con: 28.1 ± 3.4 Exp: 28.1 ± 3.5 mos	Ex_1_: Tongue ROM exercises Ex_2_: Buccinator exercises Ex_3_: Breathing exercises Ex_4_: Throat/voicing exercises	Ex_1_: × 4 reps each Ex_2_: × 4 reps each Ex_3_: × 8 reps Ex_4_: × 4 reps each	1x/day	4 wks	Waitian drinking test SSA SWAL–QOL VFSS	NC
Zhou et al. (2019) [57]	Con^a^: 30 (58.41 ± 8.65) Exp: 30 (58.54 ± 8.71)	NR	Ex_1_: Head and neck training Ex_2_: Lip exercises Ex_3_ Tongue exercises, Ex_4_: Mandibular training, Ex_5_: Pharynx training using cold stimulation Ex_6_: Swallow reflex training Ex_7_: Eating training Ex_8_: UES opening training (Mendelsohn)	Ex_1_: 10 reps × 3 sets Ex_2_: 5 s hold × 10 reps × 2–3 sets Ex_3, 4_: Not stated Ex_5_: 10–15 min Ex_6_: × 10 reps Ex_7_: 45 min meal times Ex_8_: 3–5 s hold	1x/day, 6x/wk	4 wks	VFSS scores	*g* = 4.66^b^
							BMI	*g* = 2.06^b^
							Nutritional status:	
							Albumin	*g* = 1.53^b^
							Hemoglobin	*g* = 1.05^b^
							Nitrogen	*g* = 4.69^b^
							Occurrence of aspiration pneumonia	NC

*ASHA NOMS* American Speech–Language Hearing Association’s National Outcomes Measurement System, *BMI* body mass index, *Con* control group, *CVA* cerebrovascular accident, *EAT* Eating Assessment Tool, *Ex* exercise, *Exp* experimental group, *FDS* Functional Dysphagia Scale, *FEES* Fiberoptic Endoscopic Evaluation of Swallowing, *FOIS* Functional Oral Intake Score, *g* Hedges’ *g*, *IOPI* Iowa Oral Performance Instrument, *LEDT* laryngeal elevation delay time, *MASA* Mann Assessment of Swallowing Ability, *min* minute/s, *mos* months, *n/s* not significant, *NC* not calculable, *NR* not reported, *PAS* Penetration–Aspiration Scale, *rep* repetition, *ROM* range of motion, *RSST* Repetitive Saliva Swallowing Test, *s* seconds, *SD* standard deviation, *SpO*_*2*_ oxygen saturation, *SSA* Standardized Swallowing Assessment, *SWAL–QOL* Swallowing Quality-of-Life questionnaire, *Tx* treatment, *UES* upper esophageal sphincter, *VAS* visual analogue scale, *VFSS* Videofluoroscopic Swallow Study, *vs* versus, *wk/s* week/s

^a^Intervention, dosage and effect sizes reported for the first listed group (with studies containing > 1 group)

^b^Not reported whether results were significant or not

### Study characteristics

There were 28 randomised controlled trials, eight non-randomised controlled trials, three retrospective case controls, twelve pre/post case series and three case studies. Studies were published from 2002 [38] to 2022 [46]. See Online Appendix 4 for study designs.

### Dosages of swallowing exercises

#### Exercise type

Fourteen different swallowing exercises and twelve different swallowing programs were described in the included studies. Eleven studies investigated indirect oral exercises, including lip exercises, tongue exercises and an orofacial exercise program. Twenty-eight studies investigated indirect pharyngeal exercises, including Shaker head lift, expiratory muscle strength training and chin tuck against resistance. Ten studies investigated direct swallowing exercises, most commonly the Mendelsohn manoeuvre and effortful swallow. Twelve studies examined a combined swallowing program. Some studies examined more than one intervention. The most reported exercise was Shaker head lift (investigated in ten studies). See Table 1 for single swallowing exercises in included studies (including swallowing exercises, dosages, outcome measures and effect sizes), Table 2 for combined swallowing programs and Table 3 for definitions of common exercises.

**Table 3:** Descriptions of common swallowing exercises

Swallowing exercise	Description of exercise	Target of the exercise
Indirect exercises		
Chin tuck against resistance (CTAR) [83]	A variation of the Shaker exercise that involves pressing the chin downwards against resistance in a seated position	Strengthen suprahyoid muscles to improve hyoid excursion and upper oesophageal sphincter opening
Expiratory muscle strength training (EMST) [67]	Blow with force to generate high expiratory pressures against adjustable resistance with an EMST device	Strengthen expiratory muscles and suprahyoid muscles to improve cough strength and hyoid excursion
Jaw opening exercise	Open the jaw with/without resistance	Strengthen suprahyoid muscles to improve hyoid excursion
Oral-motor or orofacial muscle exercises	Exercises involving moving oral muscles (typically lips, tongue and jaw) as far as possible, as strongly as possible with/without resistance or as quickly as possible	Improve the range-of-movement, strength and/or co-ordination of oral muscles
Shaker head lift [38]	Raise the head to look at the toes while in supine position	Strengthen suprahyoid muscles to improve upper oesophageal sphincter opening
Direct exercises		
Effortful swallow [84]	Swallow as hard as possible	Improve hyoid excursion, tongue base retraction and pharyngeal constriction
Masako [85]	Protrude the tongue and hold it between the teeth while swallowing	Improve anterior movement of the posterior pharyngeal wall
Mendelsohn maneuver [86]	Prolong the elevation of the larynx at the peak of a swallow	Increase hyolaryngeal elevation and duration of upper oesophageal sphincter opening
Supraglottic swallow [87]	Take a breath and hold it, swallow while holding the breath and cough immediately post-swallow	Improve airway protection

In general, exercise type was well-reported but varied between studies. Studies named specific exercises or provided detailed descriptions of how to conduct exercises (as per inclusion criteria). Consistent descriptions were mostly used when replicating the same exercises. For example, studies examining Shaker head lift all involved patients lifting their heads to look at their feet while in supine position. However, there was variation in devices and variation between combined exercise programs. In studies investigating single exercises, such as chin tuck against resistance, jaw opening and lip training, the main variations were in the use of devices. For example, the jaw opening exercise was described with four different types of resistance: none, against a trainer’s hand, against a jaw opening device, or against a ball. In studies investigating combined intervention programs, there was variation in which exercises were included. While all combined programs included orofacial exercises, they varied in whether they included pharyngeal exercises, swallowing with or without real boluses and breathing exercises.

#### Frequency

Frequency of intervention was well-reported but varied between studies. Frequency was consistently described by the number of sets per day and/or number of days per week of intervention. Most studies conducted intervention five times per week (30 out of 54 studies). However, frequency ranged from one to ten sets per day, and from one time per fortnight to seven times per week [31, 36].

#### Intensity

Studies varied in intensity and in how they reported intensity. All studies reported the number of repetitions of each exercise (as per inclusion criteria). Nine studies reported both number of repetitions and length of sessions [28, 34, 39–42, 70, 80, 88]. Total exercise repetitions ranged from 3 to 411 repetitions per session [48, 57, 75, 89] and from 7 to 500 repetitions per day [50, 60].

Seventeen studies reported intensity as a percentage of one-repetition maximum (with one-repetition maximum [1-RM] defined as the maximum resistance that can be applied to one exercise repetition through full range of motion) [12]. Indirect strengthening or accuracy exercises using devices—most commonly expiratory muscle strength training and tongue strengthening exercises—were more likely to report a specific intensity level. All expiratory muscle strength training studies specified exercise intensity, but targets ranged from 30% to > 70% of 1-RM. Six tongue strengthening exercises specified a target intensity of either 60% or 80% of 1-RM for strengthening and between 25% and 85% of 1-RM for accuracy training. For chin tuck against resistance, game-based biofeedback, jaw opening, Mendelsohn and Intensive Dysphagia Rehabilitation, one study per intervention specified a target intensity, e.g., 70% of 1-RM with a device.

Intensity of swallowing exercises was otherwise reported and measured in different ways. Many studies used qualitative descriptions of effort (e.g., “as hard as possible”, “to maximum extent”) [39, 73]. Biofeedback (e.g., surface electromyography) with set targets was used in four studies to set specific intensity levels [28, 66, 72, 78]. External resistance was used in around 12 studies with varying forms of resistance depending on the exercise type. Intensity or task difficulty was increased in some studies through increasing length of holds, number of repetitions or changing the amount or substance being swallowed [33, 36, 57]. See Table 4 for different ways swallowing exercise intensity was reported in studies.

**Table 4:** Ways that intensity of swallowing exercises was reported or varied in studies

Swallowing exercise	Different ways intensity was reported or varied
Chin tuck against resistance (CTAR)	Verbal description, e.g., “as far as possible” [31], “as strongly as possible” [52] Use of external resistance, e.g., device [52, 71], resistance bar [72], ball [31] Game-based feedback at certain percentage of 1-RM [72]
Effortful swallowing	Verbal description, e.g., “as hard as possible” [73], “as forcefully as possible” [62]
EMST/breathing exercises	Expiratory pressure set on device [50, 64, 65, 67] Verbal description, e.g., “deep and forceful breaths” “fast and forcefully”
Jaw opening	Against external resistance (e.g., ball, device) [54, 58, 74] Against trainer’s hand at set percentage of maximum voluntary contraction using sEMG feedback [66] Verbal description, e.g., to “maximum extent” [35]
Lip exercises	Gradually increasing external resistance, e.g., pulling force on oral screen [75], oral device [59] Percentage of maximum pressure with IOPI [76]
Mendelsohn	Increasing length of laryngeal excursion [36] Use of sEMG feedback [78] Verbal description, e.g., swallow “long and strong” [78]
PNF-based exercises	Tester providing resistance in opposite direction to jaw or neck movement [79]
Swallowing	Percentage of maximum pressure and different lengths of swallow hold using game-based biofeedback [28] Against resistance from kinesiology taping [60] Gradually increasing amount of food to be swallowed [57]
Tongue strengthening	Percentage of maximum pressure with IOPI or MOST, ranging from 60% to 80% [27, 49, 51, 68, 69] Verbal description, e.g., “press strongly” [62] or “as hard as possible” against roof of mouth [29]
Therapeutic programs	Use of external resistance, e.g., external force pushing in opposite direction [56, 82] Increasing training target goals, repetitions, or duration [33] Move from passive to active exercise and/or increasing amount of activity [57] Verbal description, e.g., “as much as possible” [39], “very hard” [34, 44]

*1-RM* 1-repetition maximum, *CTAR* chin tuck against resistance, *EMST* expiratory muscle strength training, *IOPI* Iowa Oral Performance Instrument, *MOST* Madison Oral Strengthening Therapeutic device, *PNF* Proprioceptive Neuromuscular Facilitation, *sEMG* surface electromyography

#### Time

Time, or duration, of intervention was reported as the number of days, weeks, or months of intervention. Most studies had an intervention duration of 4 weeks (22 studies), or 6 weeks (12 studies). However, across all studies, duration ranged from 6 days to 12 weeks [41, 42, 69, 70]. Three studies reported duration based on performance (e.g., once patients were no longer tube feeding) [36, 43, 63]. See Tables 1 and 2 for detailed exercise dosages reported in included studies.

#### Standard care

Around half (27) of included studies provided some form of standard care additional to their experimental intervention, either given to a control group for comparison or used as a co-intervention in both groups. Synonymous terms were used to describe this baseline intervention, including “conventional dysphagia therapy”, “traditional dysphagia therapy”, “regular” or “routine” training. While all included studies described the dosage of the experimental intervention in detail, the same level of detail was not used when describing the dosage of interventions in standard care. Most studies used general terms (e.g., orofacial muscle exercises, therapeutic manoeuvres) to describe what was involved in standard care rather than naming specific exercises. Most studies stated the length of intervention time provided in standard care groups but not the number of exercise repetitions and no other measures of intensity. Standard care was typically provided for 30 min, 5 days per week for 4 weeks. However overall, in studies that described exercises, there was variation in which exercises were included in “standard care” and in their dosages. See Table 5 for details on interventions and dosages used in “standard care” groups.

**Table 5:** Interventions and dosages of “standard care” (SC) for dysphagia used in control groups or as co-interventions

Study	Study groups	Interventions provided in standard care	Dosage of standard care		
			Intensity	Frequency	Duration
Choi et al. (2017) [47]	Shaker vs SC	Orofacial muscle exercises, thermal–tactile stimulation and therapeutic or compensatory manoeuvres	30 min	5x/wk	4 wks
Choi et al. (2020) [58]	Jaw opening exercise + SC vs Shaker + SC	Oral–facial massage, thermal–tactile stimulation, and various compensatory training exercises	30 min	5x/wk	6 wks
Eom et al. (2017) [64]	EMST + SC vs sham EMST + SC	NR	30 min	5x/wk	4 wks
Gao and Zhang (2017b) [31]	Shaker + SC vs CTAR + SC vs SC	Tongue extension and mouth exercises, such as open mouth, click teeth and swallow	10 reps each	7x/wk, 3x/day	6 wks
Guillen-Sola et al. (2017) [37]	IEMT + SC vs sham IEMT + NMES + SC vs SC	Education for self-management of dysphagia, swallowing manoeuvres, individualised oral exercises	3 h	5x/wk	3 wks
Juan et al. (2013) [27]	Case study: I-PRO therapy + SC	Swallowing-specific manoeuvres (e.g., Mendelsohn), ROM exercises	NR	NR	NR
Kim and Park (2019) [71]	Modified CTAR + SC vs SC	Oral facial massage, thermal‐tactile stimulation and various compensatory trainings	30 min	5x/wk	6 wks
Kim et al. (2017) [61]	TPRT + SC vs SC	Thermal–tactile stimulation, facial massage and various manoeuvres	NR	5x/wk	4 wks
Li et al. (2019) [56]	Balloon dilatation vs acupuncture vs balloon dilatation + acupuncture vs SC	Active lip and tongue movements and resistance exercises, ice stimulation, swallows, respiration training, masticatory muscle training, electrical stimulation	5 reps each, 10–15 swallows	5x/wk	6 wks
Liaw et al. (2020) [55]	Respiratory muscle training + SC vs SC	Postural training, breathing control, improving cough technique, checking chest wall mobility, fatigue management, orofacial exercises, thermal–tactile stimulation, Mendelsohn manoeuvre, effortful swallowing or supra-glottic manoeuvre	NR	NR	NR
Logemann et al. (2009) [32]	Shaker vs SC	Super-supraglottic swallow, Mendelsohn manoeuvre, BOT exercises (pull and hold tongue back, yawn, gargle and hold tongue in retracted position)	5 min	2x/wk (with SP), 10x/day (I)	6 wks
Mepani et al. (2009) [34]	Shaker vs SC	Falsetto, pulling tongue back in the mouth as far as possible, holding extreme yawn position, gargling, holding tongue base position. Super-supraglottic, Mendelsohn and effortful swallow	1 s hold each ex, 5 sets per day	NR	NR
Moon et al. (2017) [50]	EMST vs SC	Orofacial exercises, thermal–tactile stimulation, Mendelsohn manoeuvre, effortful swallow and supraglottic manoeuvre	30 min	5x/wk	4 wks
Moon et al. (2018) [51]	TPSAT + SC vs SC	Mendelsohn manoeuvre, effortful swallow	30 min	2x/day, 5x/wk	8 wks
Oh et al. (2017) [74]	Jaw opening exercise + SC	Orofacial muscle massage, palatal bowing stimulation, laryngeal movement and tongue exercises	30 min	5x/wk	4 wks
Park et al. (2015) [49]	Tongue strengthening + SC vs SC	NR	30 min	5x/wk	6 wks
Park et al. (2018) [52]	CTAR + SC vs SC	Orofacial muscle exercises, thermal–tactile stimulation, and therapeutic or compensatory manoeuvres	30 min	5x/wk	4 wks
Park et al. (2018) [76]	Lip strengthening + SC	Sensory stimulation, muscle strengthening	NR	NR	NR
Park et al. (2019) [72]	Game-based CTAR + SC vs Shaker + SC	Oral facial massage, thermal–tactile stimulation and various compensatory trainings (e.g., head tilting, rotation, chin tuck)	30 min per day	NR^a^	4 wks
Park et al. (2019) [62]	Effortful swallowing + SC vs saliva swallowing + SC	Orofacial muscle exercises, thermal–tactile stimulation using ice sticks, expiratory training	30 min	5x/wk	4 wks
Park et al. (2020) [89]	Resistive jaw opening + SC vs placebo + SC	Orofacial muscle exercises, thermal–tactile stimulation, and therapeutic or compensatory manoeuvres	30 min	5x/wk	4 wks
Ploumis et al. (2018) [70]	Cervical isometric exercises + SC vs sitting balance exercises + SC	Deglutition muscle strengthening and compensatory techniques	30 min per day	NR^a^	12 wks
Wei et al. (2017) [63]	Modified balloon dilatation + SC vs SC	Effortful swallow, Mendelsohn manoeuvre, supraglottic swallow and head rotation	10 reps each, 30 min	5x/wk, 2x/day	3 wks
Xing et al. (2019) [44]	Acupuncture + SC vs SC	Tongue and buccinator exercises, breathing exercises, throat muscle exercises	4 reps each (8 breaths)	NR	4 wks
Zhou et al. (2019) [57]	Nasal feeding indwelling + SC vs intermittent nasal feeding + rehab training	Ice cotton swab cold stimulation, dysphagia treatment device stimulation	30–40 min	1x/day	4 wks

*(I)* independently, *CTAR* chin tuck against resistance, *EMST* expiratory muscle strength training, *ex* exercise, *IEMT* inspiratory expiratory muscle training, *NMES* neuromuscular electrical stimulation, *NR* not reported, *rehab* rehabilitation, *reps* repetitions, *ROM* range of motion, *s* seconds, *SC* standard care, *SP* speech pathologist, *TPSAT* tongue pressure strength and accuracy training, *TPRT* tongue-to-palate resistance training, *UES* upper esophageal sphincter, *vs* versus, *wk/s* week/s

^a^Study stated 30 min per day but did not specify number of days per week

#### Reported rationales for dosage prescription

Only five studies specifically described an evidence-based rationale for their dosage. The recommended dosage for strength training drawn from limb rehabilitation research (i.e., ten repetitions, three times per day, 3 days per week for 8 weeks) [90] was applied to tongue strengthening exercises [27, 68] and effortful swallowing [62]. Two studies provided a high dosage of intervention, derived from principles of neural plasticity and/or exercise physiology [33, 78]. One crossover study investigating a high-intensity program with Mendelsohn manoeuvre showed improved outcomes on treatment weeks compared to non-treatment weeks, and with 2 weeks of intervention compared to 1 week [78]. No other studies in this review specifically compared different dosages of the same intervention.

### Outcome measures

There was a wide range of different outcome measures used. Within 54 studies, 52 different outcome measures were used. The most commonly reported outcome measure was the Penetration–Aspiration Scale [91] (used in 23 studies) which rates depth and severity at which food or drink is aspirated into the airway. The next most commonly used measures were the Functional Oral Intake Scale [92] (12 studies) which rates level of oral intake, hyoid displacement/elevation (ten studies) and maximal tongue strength (nine studies). See Tables 1 and 2 for outcome measures used in included studies.

### Risk of bias

There were 12 studies of high quality, 25 good quality and 17 low quality. Studies generally performed well on having similar groups and participants, using a valid and reasonable outcome measure, and reporting significance, effect size and confidence interval. The quality markers that were least often observed were blinding of assessors, randomised sampling, treatment fidelity and intention-to-treat analysis. See Online Appendix 4 for quality ratings for each study.

## Discussion

This systematic review found that overall, the dosages of swallowing exercises in post-stroke dysphagia studies were poorly reported, and when reported, varied significantly. Most post-stroke dysphagia studies were excluded from review due to under-reporting of exercise dosage, particularly intensity. While swallowing intervention was often provided five times per week for 4 weeks, there was a wide breadth of different exercises, frequencies, durations and intensities of intervention, with a range of different study designs and outcome measures. Variation between studies may have been due to arbitrary selection of intervention dosage, with few studies reporting a rationale for their dosage. Due to the heterogeneity of results, it was difficult to determine optimal dosages of swallowing exercises. Despite a growing awareness of the importance of dosage, more work is needed to improve consistency of dosage reporting and identify evidence-based principles to optimise prescription of swallowing exercise dosages.

To investigate dosages of swallowing exercises, we needed to identify if dosages were reported in studies. One hundred and forty-six studies were excluded due to not providing a detailed description of swallowing exercises. Only 27% of the 200 studies which matched the first three inclusion criteria (i.e., rehabilitative intervention studies in post-stroke dysphagia) reported frequency, intensity, time, or type of intervention in detail, and were included for review. Even within included studies, most studies only described the dosage of the experimental intervention in detail but not the dosage of the non-experimental or control intervention. These findings reveal an under-reporting of dosage in post-stroke dysphagia studies. Poor reporting of interventions and dosages prevents reliable implementation, comparison and replication of interventions [19]. Evidence suggests that this is an issue that extends beyond dysphagia research [93, 94]. The disproportionate under-reporting of control group interventions has also been highlighted in stroke rehabilitation studies [95]. While checklists exist to improve reporting of interventions [19, 96], specific guidelines around comprehensive dosage reporting would further improve study reporting.

Better reporting and measurement of intensity of swallowing exercises is needed. Most studies excluded for not providing a detailed description of dosage did not report intensity. Within included studies with dosage descriptions, exercise type, frequency and duration were relatively well-reported compared to intensity. Intensity is defined as how hard or how much effort an exercise involves [12]. The methods used by studies in this review may provide a starting point when considering how to measure, change and report on swallowing exercise intensity (see Table 4). Number of exercise repetitions provides some indication of intensity but does not describe amount of effort [97]. Providing both exercise repetitions and session length (as reported in nine studies) allows calculation of dosage rate, which contributes to intensity [98]. Qualitative descriptors (e.g., “as hard as you can”) or patient rating scales [99] can indicate subjective level of effort. Exercises using devices can set specific intensity levels as percentages of 1-RM, in a similar format to limb training. However, there was still significant variation in how intensity was measured, and many swallowing exercises did not have routine ways to set or measure intensity, such as a Masako manoeuvre, or head lift exercise (see Table 3 for explanations of these exercises). To properly quantify dosages, further work is needed to identify consistent methods to set and report exercise intensity for the wide range of swallowing exercises.

Examining studies that did report dosage showed significant variation in dosages of swallowing exercises in both experimental and standard care interventions. A wide range of different exercises were used, and there was variation in dosage across different swallowing exercises, similar to findings in a previous review [15]. This variation was also observed in standard care, or “conventional” or “traditional dysphagia therapy”. While a similar repertoire of exercises was used, the combination of exercises and dosages involved in standard care varied between studies. This variation in exercise selection and dosage is reflected in surveys of speech pathologists, which show no true “standard care” in dysphagia management [16]. While some variation is inherent to dysphagia intervention, using different forms of standard care in studies impacts on the ability to compare and determine relative intervention efficacies [96, 100]. Consistency in “standard care” is needed for a stable baseline in research. Evidence-based guidelines and rationales for setting intervention dosages are also needed to reduce unwarranted variation in clinical care.

Studies in this review appeared to have different rationales for dosage prescription. Only five studies specifically provided a rationale for their dosage prescription, drawn from strength-training in limb exercises and neural plasticity principles. Most commonly, studies appeared to replicate dosages from previous studies. This was seen in the Shaker exercise, where seven out of nine studies used the same 30 isotonic and three 60-s isometric head lifts used in the pioneering article [38]. Convenience also appeared to be a contributing factor. Most studies provided intervention five times per week, likely catering to typical working days rather than rehabilitation need. Overall, there was lack of evidence-based rationales for dosage prescription, similar to findings in stroke rehabilitation literature [101]. Transparent reporting of the rationale for dosage prescription in studies could facilitate better consideration of dosage selection and allow readers to understand the reasoning of researchers. The use of strength-training and neural plasticity principles offer some direction when considering rationales for swallowing exercise dosages. These principles include specificity (targeting swallow-specific exercises), increasing the volume of intervention and introducing resistive loading to swallowing exercises [102–104].

This study had several strengths and weaknesses. The strengths included the systematic approach following PRISMA guidelines. The review followed ethical guidelines, including pre-registering a protocol. The comprehensive search strategy, and inclusion of studies without limitations on language or study design, generated a high number of studies. Study selection included blinded screening and quality assessment of studies by two independent authors to reduce bias. Further, findings included measures of effect size despite the heterogeneity of results. The limitations in this systematic review were related to the quality and heterogeneity of studies. To capture all swallowing exercises and dosages used in stroke rehabilitation, all study designs and quality ratings were included and there was no specification of outcome measures. This allowed a more thorough investigation of the topic, but may have introduced biases [105]. Using a less well-known quality appraisal tool to cater for various study designs may have also impacted on quality assessment. Finally, the focus on intervention methodology rather than outcome precluded in-depth statistical analysis or data synthesis.

## Conclusions

Dosage is important in exercise-based intervention. There is increasing awareness of the importance of intentional dosage prescription and reporting in research. However, this review indicates that further work is needed to improve consistent dosage reporting and evidence-based dosage prescription in post-stroke dysphagia studies.

Uniform terminology and frameworks are needed to improve consistent and comprehensive dosage reporting across the field. Current frameworks can be used when prescribing and reporting on dosage [11, 12, 15, 106]. Methods used by studies in this review could help guide setting and reporting of intensity in clinical practice. Given the variable reporting of exercise types and dosages, clinicians should pay careful attention to the descriptions of exercises and dosages in studies when replicating or evaluating new interventions. More consistent dosage reporting within studies will improve quality and useability of studies and facilitate reproducibility, comparison, and synthesis of research.

Further work is also needed to improve evidence-based dosage prescription. Current evidence (such as strength training and neural plasticity principles) can be considered along with clinical reasoning to guide dosage prescription. However, more research is needed to examine which principles are applicable to dysphagia rehabilitation. Future research could investigate the impact of altering different components of dosage, such as comparing similar interventions provided at different dosages. Improved dosage reporting, and evidence-based dosage prescription has the potential to improve intervention efficacy and outcomes for patients with post-stroke dysphagia.

## Supplementary Information

Below is the link to the electronic supplementary material.

Supplementary file1 (DOCX 41 KB)

## Data Availability

The data that support the findings of this study are available from the corresponding author upon reasonable request.
