# Supplementary material for: Dosages of swallowing exercises in stroke rehabilitation: a systematic review
Source: Eur Arch Otorhinolaryngol. 2022 Dec 6;280(3):1017–45. doi: 10.1007/s00405-022-07735-7 (PMC9899761; doi:10.1007/s00405-022-07735-7)
Supplement: Supplementary file 1 — Supplementary file1 (DOCX 41 KB) [file 405_2022_7735_MOESM1_ESM.docx]

**Appendix 1** Full search strategy used for MEDLINE, Embase via Ovid and CINAHL

| 1. Deglutition/ |
| --- |
| 2. Deglutiti*.mp. |
| 3. Deglutition Disorders/ |
| 4. Deglutition Disorder*.mp. |
| 5. swallow*.mp. |
| 6. swallowing disorder*.mp. |
| 7. dysphagi*.mp. |
| 8. ((swallow* or deglutit* or dysphag*) adj5 (disturbance* or disorder* or difficult* or dysfunction* or impair* or condition* or abnormal* or damage* or injur*)).tw. |
| 9. Pharynx/ |
| 10. Pharyn*.mp. |
| 11. Pharyngeal Muscles/ |
| 12. pharyngeal musc*.mp. |
| 13. ((pharyn* or oropharyn*) adj3 (disturbance* or disorder* or difficult* or dysfunction* or impair* or condition* or abnormal* or damage* or injur*)).tw. |
| 14. 1 or 2 or 3 or 4 or 5 or 6 or 7 or 8 or 9 or 10 or 11 or 12 or 13 |
| 15. exp Stroke/ |
| 16. Stroke*.mp. |
| 17. Cerebrovascular Disorders/ |
| 18. cerebrovascular disorder*.mp. |
| 19. cerebrovascular accident*.mp. |
| 20. CVA.mp. |
| 21. ((brain* or cerebr* or cerebell* or vertebrobasil* or hemisphere* or intracran* or intracerebral or infratentorial or supratentorial or middle cerebral artery or MCA* or anterior circulation or posterior circulation or basilar artery or vertebral artery) adj5 (isch?emi* or infarct* or thrombo* or emboli* or occlus* or accident)).tw. |
| 22. (stroke* or poststroke or apoplexy* or cerebral vasc* or brain vasc* or cerebrovasc* or cva*).tw. |
| 23. ((brain* or cerebr* or cerebell* or intracerebral or intracran* or parenchymal or intraparenchymal or intraventricular or infratentorial or supratentorial or basal gangli* or putaminal or putamen or posterior fossa or hemispher*) adj5 (h?emorrhag* or h?ematoma* or bleed*)).tw. |
| 24. 15 or 16 or 17 or 18 or 19 or 20 or 21 or 22 or 23 |
| 25. Exercise/ |
| 26. Exercise*.mp. |
| 27. Exercise Therapy/ |
| 28. Exercise Therap*.mp. |
| 29. Swallow* therap*.mp. |
| 30. Shaker.mp. |
| 31. Mendelsohn.mp. |
| 32. Masako.mp. |
| 33. chin tuck.mp. |
| 34. Neurological Rehabilitation/ |
| 35. Stroke Rehabilitation/ |
| 36. Rehabilitation/ |
| 37. rehabilitat*.mp. |
| 38. Iowa Oral Performance Instrument.mp. |
| 39. Madison Oral Strengthening Therapeutic.mp. |
| 40. TheraBite.mp. |
| 41. or?motor.mp. |
| 42. CTAR.mp. |
| 43. jaw open*.mp. |
| 44. recline exercise.mp. |
| 45. head lift.mp. |
| 46. head lift exercise.mp. |
| 47. chin to chest.mp. |
| 48. open swallow*.mp. |
| 49. effortful swallow.mp. |
| 50. ((swallow* or dysphagi* or deglutition* or oropharyn* or pharyn*) adj5 (therap* or exercis* or rehabilit*)).tw. |
| 51. McNeill Dysphagia Therapy Program.mp. |
| 52. head extension.mp. |
| 53. Lee Silverman Voice Treatment.mp. |
| 54. 25 or 26 or 27 or 28 or 29 or 30 or 31 or 32 or 33 or 34 or 35 or 36 or 37 or 38 or 39 or 40 or 41 or 42 or 43 or 44 or 45 or 46 or 47 or 48 or 49 or 50 or 51 or 52 or 53 |
| 55. 14 and 24 and 54 |

*indicating truncated term was used

**Appendix 2** Full search strategy used for Web of Science

| 1. (((((ALL=(Deglutiti*)) OR ALL=("Deglutition Disorder*")) OR ALL=(swallow*)) OR ALL=("swallowing disorder*")) OR ALL=(dysphagi*)) OR ALL=(Pharyn*) |
| --- |
| 2. ((((((((ALL=(Stroke*)) OR ALL=("Cerebrovascular Disorder*")) OR ALL=("cerebrovascular accident*")) OR ALL=(poststroke)) OR ALL=(apoplexy*)) OR ALL=(cerebral vasc*)) OR ALL=(brain vasc)) OR ALL=(cerebrovasc*)) OR ALL=(CVA*) |
| 3. ((((((((((((((((((((((((ALL=(Exercis*)) OR ALL=("Exercise Therap*")) OR ALL=("Swallow* therap*")) OR ALL=(Shaker)) OR ALL=(Mendelsohn)) OR ALL=(Masako)) OR ALL=("chin tuck")) OR ALL=("Neuro* Rehabilitation")) OR ALL=("Stroke Rehabilitation")) OR ALL=(Rehabilitat*)) OR ALL=("Iowa Oral Performance Instrument")) OR ALL=("Madison Oral Strengthening Therap*")) OR ALL=(TheraBite)) OR ALL=(or?motor)) OR ALL=(CTAR)) OR ALL=("jaw open*")) OR ALL=("recline exercise")) OR ALL=("head lift")) OR ALL=("head lift exercise")) OR ALL=("chin to chest")) OR ALL=("open swallow")) OR ALL=("effortful swallow")) OR ALL=("McNeil Dysphagia Therapy Program")) OR ALL=("head extension")) OR ALL=("Lee Silverman Voice Treatment") |
| 4. 1. AND 2. AND 3. |

*indicating truncated term was used

**Appendix 3** Full search strategy used for SpeechBITE

| **Advanced search** | **Option selected** |
| --- | --- |
| Speech Pathology Practice Area: | Dysphagia |
| Type of intervention: | Swallowing/feeding intervention |
| Within this population: | Stroke/CVA |
| Age group: | Adults |

**Appendix 4** Study designs and quality ratings of included studies (ASHA Levels of Evidence Tool)

| **Study** | **Study design** | **Blind-ing** | **Random sampling** | **Group/ participant comparability** | **Treatment fidelity** | **Outcomes** | **Signifi-cance** | **Precision** | **Intent-to-treat** | **Quality Score** |
| --- | --- | --- | --- | --- | --- | --- | --- | --- | --- | --- |
| Arnold et al. (2020) | Retrospective case control | No | No | Yes | No | Yes | Yes | Yes | N/A | 4 |
| Bogaardt et al. (2009) | Retrospective case control | No | No | Yes | No | Yes | Yes | Yes | N/A | 4 |
| Cho et al. (2017) | Case series | No | No | No | No | Yes | Yes | Yes | N/A | 3 |
| Choi et al. (2017) | Controlled trial | No | No | Yes | Yes | Yes | Yes | Yes | No | 5 |
| Choi et al. (2020) | Controlled trial | No | Yes | Yes | No | Yes | Yes | Yes | No | 5 |
| Oh et al. (2017) | Case series | No | No | No | No | Yes | No | No | N/A | 1 |
| Oh et al. (2013) | Controlled trial | No | Yes | No | Yes | Yes | Yes | Yes | No | 5 |
| El-Tamawy et al. (2015) | Controlled trial | Yes | Yes | Yes | Yes | Reasonable | Yes | Yes | Yes | 7 |
| Eom et al. (2017) | Controlled trial | Yes | Yes | Yes | Yes | Yes | Yes | Yes | No | 7 |
| Gao et al. (2017) | Controlled trial | No | Yes | Yes | No | Yes | Yes | Yes | Yes | 6 |
| Guillen-Sola et al. (2017) | Controlled trial | Yes | Yes | Yes | Yes | Yes | Yes | Yes | Yes | 8 |
| Hagg et al. (2008) | Retrospective case control | No | No | No | No | Yes | Yes | No | N/A | 2 |
| Hagglund (2020) | Controlled trial | Yes | Yes | Yes | Yes | Yes | Yes | Yes | Yes | 8 |
| Hamzic (2021) | Case study | No | No | Yes | No | Yes | No | No | No | 2 |
| **Study** | **Study design** | **Blind-ing** | **Random sampling** | **Group/ participant comparability** | **Treatment fidelity** | **Outcomes** | **Signifi-cance** | **Precision** | **Intent-to-treat** | **Quality Score** |
| Hegland et al. (2016) | Case series | Yes | No | Yes | Yes | Yes | Yes | Yes | N/A | 6 |
| Moon et al. (2017) | Controlled trial | No | No | Yes | Yes | Yes | Yes | Yes | Yes | 6 |
| Jiao (2022) | Controlled trial | No | Yes | Yes | No | Yes | Yes | No | Yes | 5 |
| Juan et al. (2013) | Case study | Yes | No | Yes | No | Yes | No | No | N/A | 3 |
| Jung (2020) | Controlled trial | Yes | No | Yes | No | Reasonable | Yes | Yes | No | 4 |
| Kim & Park (2019) | Controlled trial | No | Yes | Yes | No | Yes | Yes | Yes | No | 5 |
| Kim et al. (2010) | Case series | No | No | Yes | Yes | Reasonable | Yes | Yes | N/A | 4 |
| Kim et al. (2015) | Controlled trial | No | No | Yes | Yes | Yes | Yes | Yes | Yes | 6 |
| Kim et al. (2017) | Controlled trial | No | Yes | Yes | Yes | Yes | Yes | Yes | No | 6 |
| Koyama et al. (2017) | Controlled trial | Yes | Yes | Yes | Yes | No | Yes | Yes | No | 6 |
| Krajczy et al. (2019) | Controlled trial | No | Yes | Yes | No | Reasonable | Yes | Yes | Yes | 5 |
| Kumaresan et al. (2018) | Case series | No | No | No | No | Yes | Yes | Yes | Yes | 4 |
| Li et al. (2019) | Controlled trial | No | Yes | Yes | No | Yes | Yes | Yes | Yes | 6 |
| Liaw et al. (2020) | Controlled trial | Yes | Yes | Yes | Yes | Yes | Yes | Yes | Yes | 8 |
| Logemann et al. (2009) | Controlled trial | Yes | No | Yes | Yes | Yes | Yes | Yes | No | 6 |
| Malandraki et al. (2016) | Case series | Yes | No | Yes | Yes | Yes | Yes | Yes | Yes | 7 |
| McCullough et al. (2013) | Controlled trial | No | Yes | Yes | Yes | Yes | Yes | Yes | No | 6 |
| Mepani et al. (2009) | Controlled trial | Yes | No | Yes | Yes | Reasonable | Yes | Yes | No | 5 |
| **Study** | **Study design** | **Blind-ing** | **Random sampling** | **Group/ participant comparability** | **Treatment fidelity** | **Outcomes** | **Signifi-cance** | **Precision** | **Intent-to-treat** | **Quality Score** |
| Momosaki et al. (2014) | Case series | No | No | Yes | Yes | Yes | No | Yes | N/A | 4 |
| Momosaki et al. (2015) | Case series | No | No | Yes | Yes | Yes | Yes | Yes | N/A | 5 |
| Moon et al. (2018) | Controlled trial | Yes | Yes | Yes | No | Yes | Yes | Yes | Yes | 7 |
| Moon et al. (2019) | Controlled trial | No | No | Yes | Yes | Yes | Yes | Yes | Yes | 6 |
| Park et al. (2015) | Controlled trial | No | Yes | Yes | Yes | Yes | Yes | Yes | No | 6 |
| Park et al. (2016) | Controlled trial | Yes | Yes | Yes | Yes | Yes | Yes | Yes | No | 7 |
| Park et al. (2017) | Controlled trial | No | No | Yes | No | Yes | Yes | Yes | No | 4 |
| H. Park et al. (2018) | Case series | No | No | No | No | Reasonable | Yes | Yes | N/A | 2 |
| J. Park et al. (2018) | Controlled trial | No | Yes | Yes | Yes | Yes | Yes | Yes | No | 6 |
| Park et al. (2019a) | Controlled trial | Yes | Yes | Yes | Yes | Yes | Yes | Yes | No | 7 |
| Park et al. (2019b) | Controlled trial | Yes | Yes | Yes | Yes | Yes | Yes | Yes | No | 7 |
| Park et al. (2020) | Controlled trial | Yes | Yes | Yes | No | Yes | Yes | Yes | No | 6 |
| Ploumis et al. (2018) | Controlled trial | Yes | Yes | Yes | Yes | Yes | Yes | Yes | Yes | 8 |
| Robbins et al. (2007) | Case series | No | No | Yes | Yes | Yes | Yes | Yes | N/A | 5 |
| Shaker et al. (2002) | Controlled trial | Yes | Yes | Yes | No | Reasonable | Yes | Yes | No | 5 |
| Steele et al. (2016) | Controlled trial | Yes | Yes | Yes | No | Yes | Yes | Yes | No | 6 |
| Stepp et al. (2011) | Case study | No | No | Yes | Yes | No | No | No | N/A | 2 |
| Wada et al. (2012) | Case series | Yes | No | Yes | No | Reasonable | Yes | Yes | N/A | 4 |
| **Study** | **Study design** | **Blind-ing** | **Random sampling** | **Group/ participant comparability** | **Treatment fidelity** | **Outcomes** | **Signifi-cance** | **Precision** | **Intent-to-treat** | **Quality Score** |
| Wei et al. (2017) | Controlled trial | Yes | Yes | Yes | No | Yes | Yes | Yes | Yes | 7 |
| Xing et al. (2019) | Controlled trial | No | Yes | Yes | No | Yes | Yes | No | No | 4 |
| Yeates et al. (2008) | Case series | No | No | Yes | Yes | Yes | No | No | N/A | 3 |
| Zhou et al. (2019) | Controlled trial | No | Yes | Yes | No | Yes | Yes | Yes | Yes | 6 |

ASHA = American Speech-Language-Hearing Association; N/A = not applicable
Note: Controlled trials which received a “Yes” for Random Sampling were randomised controlled trials
